# High-resolution mapping of reciprocal translocation breakpoints using long-read sequencing

**DOI:** 10.1016/j.mex.2019.10.028

**Published:** 2019-10-31

**Authors:** Judy F.C. Chow, Heidi H.Y. Cheng, Estella Y.L. Lau, William S.B. Yeung, Ernest H.Y. Ng

**Affiliations:** a Department of Obstetrics and Gynecology, The University of Hong Kong, Queen Mary Hospital, Pokfulam, Hong Kong; b Department of Obstetrics and Gynecology, Queen Mary Hospital, Hong Kong; c Shenzhen Key Laboratory of Fertility Regulation, The University of Hong Kong-Shenzhen Hospital, Hong Kong

**Keywords:** Translocation breakpoint mapping using nanopore sequencing, Nanopore sequencing, Breakpoint, PGT-SR

## Abstract

Long-read nanopore sequencing enables direct high-resolution breakpoint mapping on balanced carriers of reciprocal translocation. The mean sequencing depth on the translocated chromosomes to achieve accurate mapping of breakpoints ranged from 2.5-fold to 6.2-fold. To speed up determination of the breakpoints from long-read sequencing data, alignment reads on the translocated chromosomes were extracted before piped into NanoSV. Checking the position of breakpoints on Interactive Genomics Viewer (IGV) was crucial to successful design of breakpoint PCR primers, especially when large deletion was involved at the breakpoints.

• Long-read sequencing enables accurate breakpoint mapping with base-pair resolution
• Splitting bam files by translocated chromosomes drastically speeded up the breakpoint determination
• IGV helps to identify the breakpoint positions and facilitate the design of breakpoint PCR primers

## Graphical abstract

**Figure:**
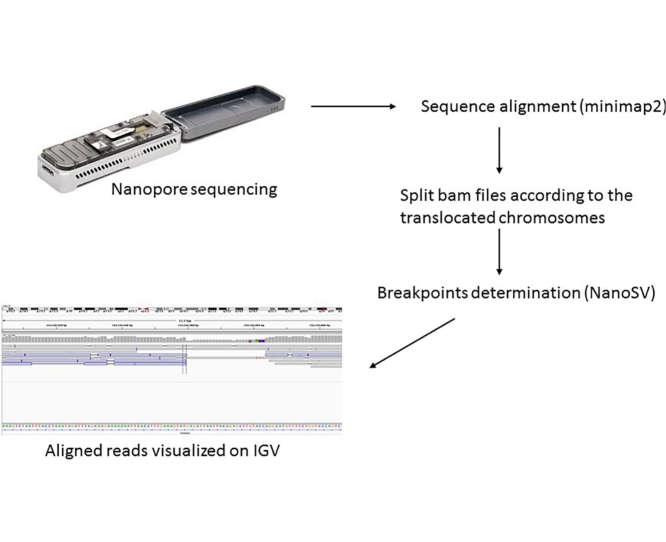

**Specification Table**

**Table:** 

Subject Area:	Biochemistry, Genetics and Molecular Biology
More specific subject area:	Preimplantation genetic testing
Method name:	Translocation breakpoint mapping using nanopore sequencing
Name and reference of original method:	Cretu Stancu, M. et al. Mapping and phasing of structural variation in patient genomes using nanopore sequencing. Nat. Commun. 8, 1326 (2017).
Resource availability:	https://github.com/mroosmalen/nanosv http://software.broadinstitute.org/software/igv/

## Method details

Genomic DNA of balanced reciprocal translocation carriers was sequenced in a MinION flow cell (R9.4, Oxford Nanopore Technologies, UK) with a 48 -h sequencing protocol [1]. In brief, genomic DNA was extracted from peripheral blood using QIAamp DNA Blood Mini kit (Qiagen, Manchester, UK). The integrity of the DNA was confirmed by gel electrophoresis. One microgram of DNA was used to prepare a sequencing library using the SQK-LSK108 kit (Oxford Nanopore, Oxford, UK). Local base calling, sequence alignment and breakpoints determination were performed, followed by confirmation of breakpoints by Sanger sequencing.

## Base calling, sequencing alignment and breakpoints determination

1) Local base calling was performed using Albacore 2.3.1 (Oxford Nanopore, Oxford, UK). FastQ files were sorted into pass and fail folders using a default quality score cutoff of 7. Please note that at the time of manuscript revision, FAST5 files generated by the updated version of MinKNOW (core 3.5.5, Oxford Nanopore, Oxford, UK), are no longer supported by Albacore. Real time basecalling can be performed by the data processing toolkit (Guppy) integrated in MinKNOW, or performed locally using Guppy Basecalling Software (Version 3.3.0).

read_fast5_basecaller.py --flowcell FLO-MIN106 --kit SQK-LSK108 --output_format fastq --input *folder_with_fast5*

--recursive –save_path fastq/ --worker_threads 12

1) The FastQ files in the “pass” folder were aligned to GRCh37/hg19 using minimap2(2.11) [2] and then converted to sorted bam files by samtools

minimap2 –ax map-ont *reference*.fasta fastq/pass/*.fastq > *example*.sam

samtools view –bS *example*.sam > *example*.bam

samtools sort *example*.bam *example*.sorted

samtools index *example*.sorted.bam

1) The bam files were split according to the translocated chromosomes. Merge of the 2 split bam files was necessary before breakpoint determination. This could significantly cut down the time for breakpoint determination. Take sample #100648 as an example, the time required was reduced 60 % after splitting the bam file.

samtools view –hb *example*.sorted.bam chrA > chrA.bam (where chrA and chrB are the translocated chromosomes)

samtools sort chrA.bam chrA.sorted

samtools view –hb *example*.sorted.bam chrB > chrB.bam

samtools sort chrB.bam chrB.sorted

samtools merge chrA_chrB.sorted.bam chrA.sorted.bam chrB.sorted.bam

samtools index chrA_chrB.sorted.bam

1) Breakpoints determination was performed using NanoSV1.2.0 [3], and all the putative breakpoints were listed in *example*.vcf

NanoSV chrA_chrB.sorted.bam –t 8 –b *reference*.bed –o *example*.vcf

**Fig. 1:**
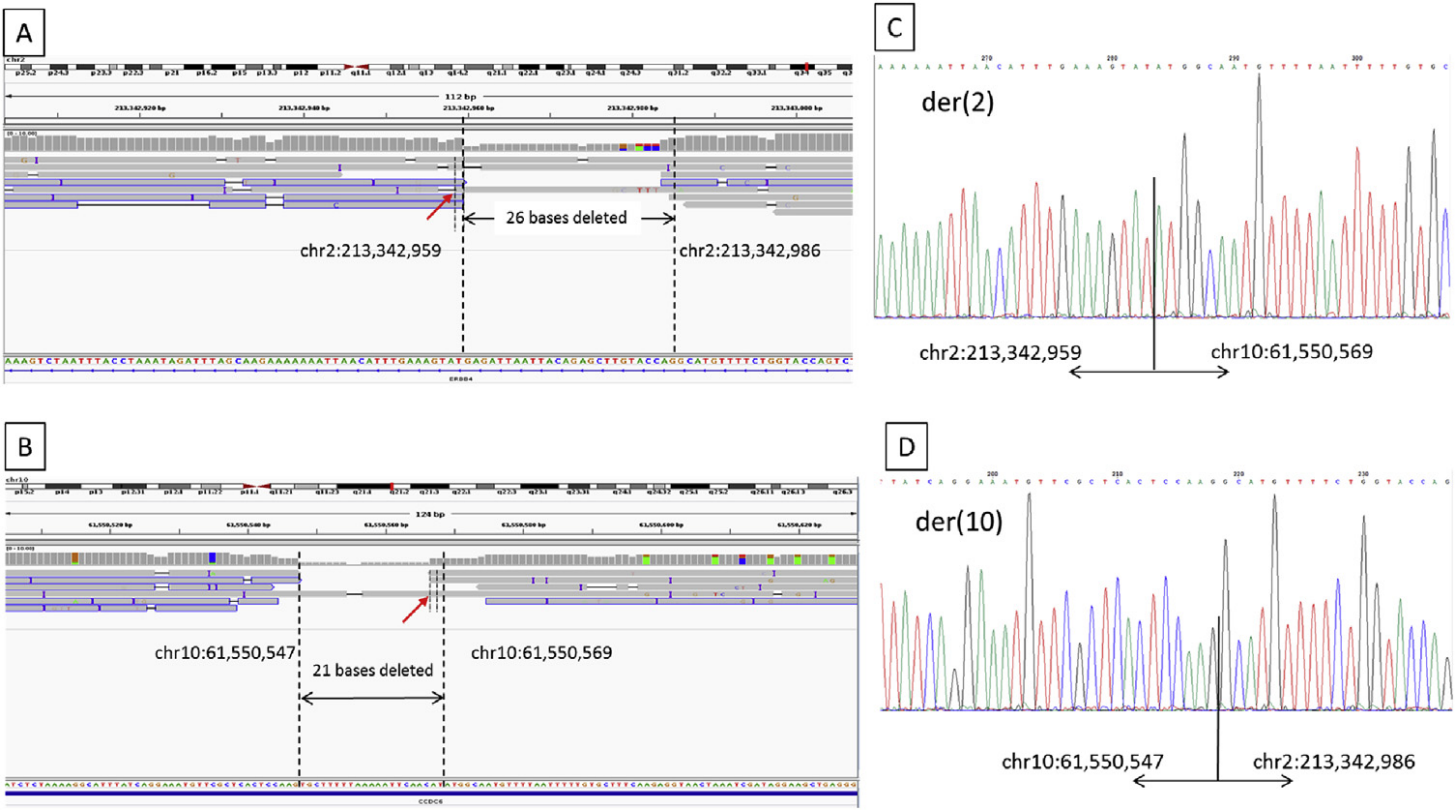
Aligned reads shown on IGV and Sanger sequencing results of derivative chromosomes of sample #100238. (A) Aligned reads on chromosome 2. (B) Aligned reads on chromosome 10. (C–D) Sanger sequencing results on der(2) and der (10) respectively, showing highly concordant results with IGV views. Red arrow indicates the breakpoints predicted by NanoSV.

Coordinates of the predicted breakpoints that were correlated with the cytogenetic reports of the patients were extracted from the *example*.vcf. Table 1 shows the extent of concordance of the predicted breakpoints of 9 samples with Sanger sequencing results. There were discrepancies of a few bases due to microinsertions / microdeletions in all samples. To get more information on the characteristics at the break junctions, positions of the breakpoints were visually checked on IGV. The aligned sequencing reads of chr2 and chr10 of sample #100238 on IGV are depicted in Fig. 1A and B. Microdeletions were easily identified at the regions showing reduced coverage between the 2 breakpoints. Sanger sequencing results of the derivative chromosomes (Fig. 1C and D) confirmed the microdeletions.

**Table 1:** Concordance of NanoSV prediction and breakpoints confirmed by Sanger sequencing.

Lab ID	Predicted breakpoints		Confirmed breakpoints (by Sanger sequencing)	
100238	chr2:213,342,959	chr10:61,550,567	chr2:213,342,959	chr10:61,550,547
100364	chr2:954,995	chr10:11,381,382	chr2:954,994	chr10:11,381,389
100573	chr7:45,828,646	chr13:32,209,016	chr7:45,828,644	chr13:32,209,008
100585	chr1:36,518,550	chr13:34,909,448	chr1:36,518,551	chr13:34,909,448
100604	chr1:234,634,530	chr18:38,268,290	chr1:234,634,529	chr18:38,268,286
100648	chr8:135,156,957	chr10:128,448,174	chr8:135,156,792	chr10:128,448,174
100784	chr9:148,32,282	chr15:48,536,822	chr9:14,832,281	chr15:48,536,811
100847	chrX:138,761,317	chr5:43,325,536	chrX:138,761,303	chr5:43,325,544
100881	chr1:238,220,865	chr5:8,479,913	chr1:238,220,864	chr5:8,479,912

**Fig. 2:**
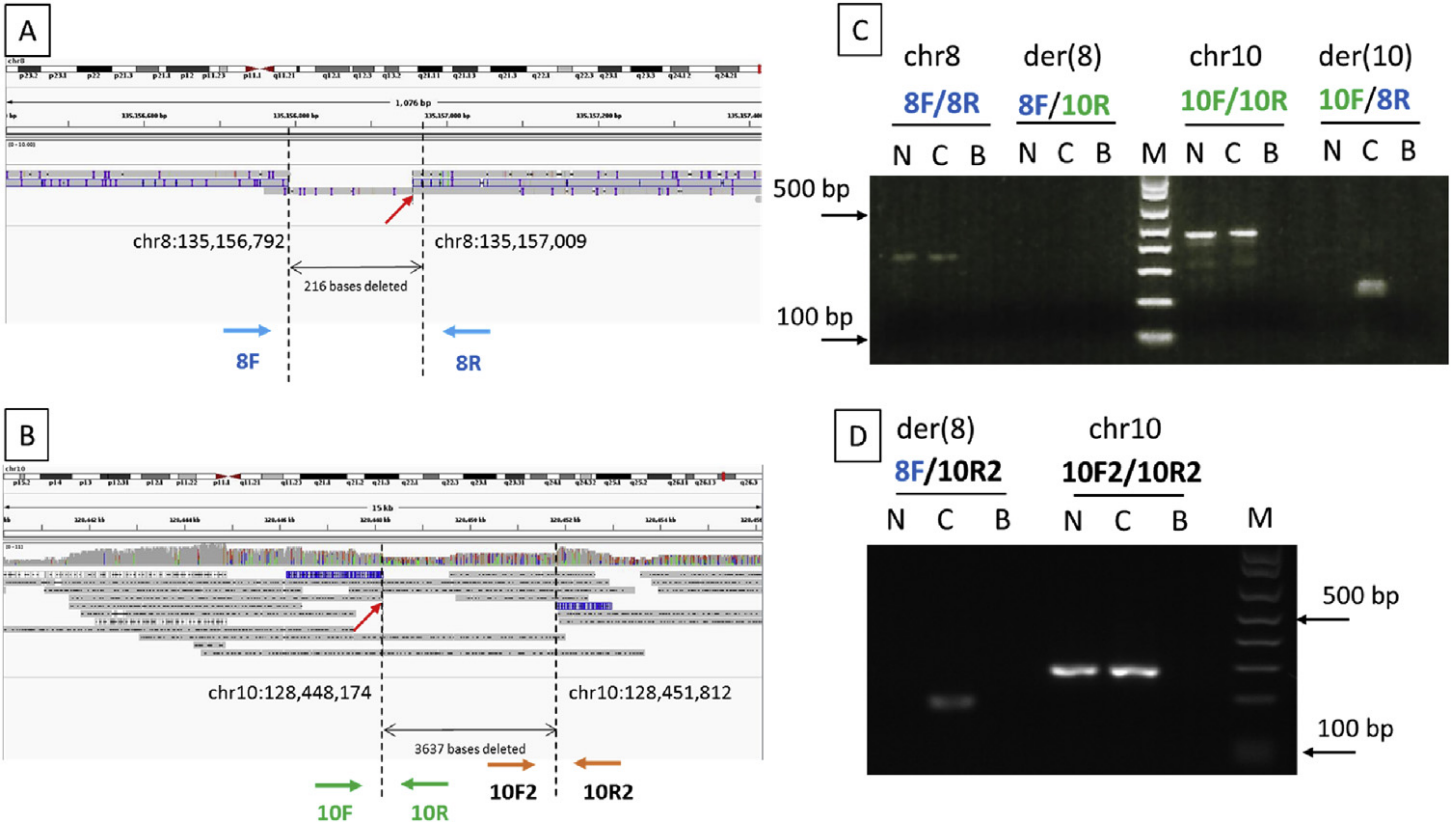
Aligned reads shown on IGV and breakpoint PCR results of sample #100648. (A) Aligned reads on chromosome 8. (B) Aligned reads on chromosome 10. Red arrow indicates breakpoints predicted by NanoSV. (C) First breakpoint PCR results. Primers on chromosome 10 were designed (10 F and 10R) solely based on the predicted breakpoint (chr10:128,448,174). (D) Second breakpoint PCR results. Primer were designed (10 F2 and 10R2) based on the chimeric read (depicted in blue box) located at the 3′ end of predicted breakpoint, visualized on IGV. N: noncarrier; C: translocation carrier; B: negative control; M: DNA molecular weight marker.

In case where a large deletion was suspected at the breakpoints, it is important to visually check the alignment reads by zooming out on IGV. For instance, the aligned reads on chr8 of sample #100648 indicated a deletion of 216 bases at the breakpoint (Fig. 2A). This provided clues to design PCR primers that covered the deletions. Fig. 2B shows the aligned reads on chr10. Primers were initially designed based on the predicted breakpoint at chr10:128,448,174. Breakpoint PCR successfully amplified chr8, chr10 and der(10) but not der(8) (Fig. 2C). It was suspected that a large deletion was involved at the breakpoint on chromosome 10 based on the following reasons. First, successful amplification of chr8 and chr10 indicated that the primers 8 F, 8R, 10 F and 10R were working. Second, successful amplification of der(10) confirmed the 5′ location of the deletion on chr10 and the 3′ location of the deletion on chr8. Third, IGV view on chromosome 8 clearly showed a deletion of approximately 200 bases between primers 8 F and 8R. Therefore, it was deduced that primer 8 F should be able to bind to der(8), and that the failure in amplification of der(8) was due to the deletion which removed the binding sequence of primer 10R. Fig. 2B shows the chimeric reads (reads aligned to both chromosome 8 and chromosome 10) at 3637 bases downstream from the predicted breakpoint (depicted in the blue box) and the 3′ location of the deletion on chromosome 10. New PCR primers flanking the 3′ end of the deletion could amplify the der(8) successfully, confirming the breakpoints on chromosome 8 and chromosome 10.

In summary, we demonstrate a new method for accurately mapping of the reciprocal translocation breakpoints directly on balanced carriers. Splitting the bam files according to the translocated chromosomes before breakpoint prediction can dramatically cut down the time required for the prediction. Visual inspection of the aligned reads on IGV is crucial to accurate identification of the breakpoints. Nonetheless, this method has a few limitations. First, it relies on the previous cytogenetic diagnosis for splitting bam files, therefore it is not applicable for mapping genome-wide de novo structural variations. Second, the method is not applicable to patients with translocation breakpoints located across the highly repetitive centromeric regions of acrocentric chromosomes, such as Robertsonian translocation carriers.

## Additional information

Balanced reciprocal translocation carriers are usually phenotypically normal but are at an increased risk of infertility, recurrent pregnancy loss or having children with physical or mental abnormalities. Preimplantation genetic testing on chromosomal structural rearrangement (PGT-SR) offers a way to screen against unbalanced embryos during in vitro fertilization treatment cycle, but the methods cannot distinguish euploid carrier from noncarrier embryos. Although replacing carrier embryos should result in phenotypically normal live births, the offspring will encounter the same problems as their parent in terms of infertility, recurrent pregnancy loss or having affected children. Distinguishing between carrier and noncarrier embryos is possible by SNP haplotyping [4,5] and next generation sequencing (NGS) [[6], [7], [8]], however these methods involved complicated analyses and interpretations. SNP haplotyping approach also relies on the presence of an unbalanced embryo, DNA of previously affected pregnancies or DNA of close relatives for phasing. Instead nanopore sequencing can provide simple, accurate and high-resolution breakpoint mapping directly on balanced carriers. This enables the design of breakpoint PCR primers which can be used to distinguish carrier from noncarrier embryos produced in PGT-SR cycles. This method allows patients to prioritize the replacement of euploid noncarrier embryos.

## Declaration of Competing Interest

The authors declare that they have no known competing financial interests or personal relationships that could have appeared to influence the work reported in this paper.
